# Anaesthetic practices at Gulu Regional Referral Hospital in Northern Uganda, who does what and where? A retrospective study

**DOI:** 10.1186/s12960-025-00987-4

**Published:** 2025-04-14

**Authors:** N. Kutschke, J. Lampe, O. Hoepfner, D. L. Kitara, A. Schuster

**Affiliations:** 1 Werner Forßmann Klinikum, Akademisches Lehrkrankenhaus der Charité, Klinik für Anästhesie und Intensivmedizin, Eberswalde, Germany; 2 Charité – University Medicine Berlin, Corporate Member of Freie Universität Berlin, Humboldt-Universität zu Berlin and Berlin Institute of Health, Berlin, Germany; 3 Gulu University and Gulu Regional Referral Hospital - Teaching Hospital of Gulu University – Faculty of Medicine, Department of Surgery, Gulu, Uganda; 4 Charité – University Medicine Berlin, Corporate Member of Freie Universität Berlin, Humboldt-Universität zu Berlin and Berlin Institute of Health, Institute of General Practice, Charitéplatz 1, 10117 Berlin, Germany

**Keywords:** Peripheral regional anaesthesia, Task division, Gulu Hospital, Northern Uganda

## Abstract

**Background:**

Hospitals such as the Gulu Regional Referral Hospital (GRRH) in northern Uganda, like many other regions of sub-Saharan Africa, lack the anaesthetists needed to provide adequate analgesia during surgical procedures.

The GRRH has not employed any anaesthesiologist for many years. Instead, anaesthesia is carried out by non-physician anaesthetic officers (AO) and other healthcare workers (HWs). In this setting, peripheral regional anaesthesia (pRA) is a safe and resource-efficient alternative that HWs and AOs could use. The study aimed to evaluate surgical procedures, anaesthetic practices, and staffing at Gulu Regional Referral Hospital in Northern Uganda. The objective was to identify the appropriate audience for pRA training and the corresponding training content.

**Methods:**

A retrospective review was conducted on surgical procedures and their anaesthetic management in three departments of GRRH during 2019. The possibility of performing pRA was determined based on the surgical site, infection status, and the type of surgical procedure being performed. A pRA was considered adequate when conditions for pRA were met and pRA was carried out. Chi-square test was used to compare categorical data. A bivariable logistic regression analysis was performed to identify the factors associated with the administration of peripheral regional anaesthesia and the qualifications of medical staff.

**Results:**

A total of 804 procedures were recorded [67% in accident and emergency (A&E), 31% in operating room (OR), and 2% on the surgical ward]. Anaesthesia was recorded in 82% of cases. Of these, 86% were documented in regional and local anaesthesia. Anaesthetic officers carried out anaesthesia in 20% of all cases and in all cases in the operating room. HWs with more than 2 years of training performed adequate pRA more frequently than HWs with less than 1 year of experience [Odds ratio (OR) = 2.586; 95% CI 1.336–5.005; *p* = 0.005]. The last group, however, performed significantly more procedures in A&E than in other departments (89%, *p* < 0.001).

Of the 209 procedures that could have been performed with pRA, 85 were found to be inadequately anaesthetised. 79% (67) of these were performed in the emergency department. In 45% of cases with inadequate anaesthesia, patients received local anaesthesia instead of appropriate pRA. Pain control was absent in 18% of cases, and 20% of cases received presumably unnecessary general anaesthesia or sedation. In 17% of cases, additional administration of ketamine and/or midazolam was required due to insufficient pRA.

**Conclusions:**

The data show that pRA procedures are already used at GRRH, especially by HWs with high level of training in the OR. In A&E, which is primarily staffed by doctors with less than 1-year training, there is a potential to increase the administration of adequate pain relief by implementing simple nerve blocks into routine clinical practice. Therefore, doctors and staff in A&E would benefit from needs-based training in pRA.

**Supplementary Information:**

The online version contains supplementary material available at 10.1186/s12960-025-00987-4.

## Introduction

Worldwide, five billion people do not have access to timely anaesthetic care. Low-income countries (LICs) which are home to almost half of the world's population, are particularly disadvantaged, as they have access to only 15.0% of the world's anaesthesiologists [1].

In 2018, the World Health Organization (WHO) and World Federation of Societies of Anaesthesiologists (WFSA) have set international standards for safe practice of anaesthesia worldwide. They estimated that the minimum number of a specialist surgical workforce (surgeons, anaesthesiologists, obstetricians) should be at least 20 per 100,000 population by 2030 [2]. Although they don´t specify the exact number of anaesthesia providers within this specialist workforce, they declare Uganda understaffed in this area, with only 0.2 physician anaesthesia providers per 100,000 population (total: 47 Anaesthesiologists and 72 non-specialist physician anaesthesia providers) according to the WFSA Workforce Map [3]. Other authors of a 2017 study recommend at least 5 physician anaesthesia providers per 100,000 population in LICs to ensure effective anaesthesia service delivery [4]. It should be noted, however, that this calculation does not include the 430 nurse anaesthetists and other non-physician anaesthesia providers in Uganda who hold a diploma in anaesthesia. Collectively, they already constitute the largest part of the anaesthetic workforce and are also known as anaesthetic officers (AO) [3, 4]. If they are included, Uganda had an anaesthesia workforce of 1.3 physician and non-physician anaesthesia providers per 100,000 population at that time, according to a 2017 survey [4]. In comparison with Germany, there are 31 anaesthetic care providers per 100,000 patients [3]. As with many other low- and middle-income countries (LMICs), political steering processes have been implemented in Uganda to address the shortage of physician anaesthesia providers. This has led to the introduction of task-sharing arrangements. Anaesthetic officers (AOs) are non-physician anaesthesia providers in Uganda who have undergone 2 years of anaesthesia training. They play a significant role in providing anaesthetic care, administering spinal and some general anaesthesia, and providing perioperative and postoperative care [5]. To become an AO, it is necessary to hold a diploma in either nursery, midwifery, environmental health science or medical laboratory techniques. This excludes many other health workers. However, the task-sharing approach could also involve other healthcare workers (HW), such as interns, nurses without further diploma and other physicians to alleviate the burden on the anaesthesia workforce and close the gap.

It is important to note that modern anaesthesia has enabled surgery to reach its current standard [6]. Invasive procedures have become more feasible due to improved monitoring, cardiovascular and ventilatory support, and adequate intraoperative pain relief [7, 8]. In general, anaesthetic care during any surgical procedure can be provided in three main ways: general anaesthesia (the medically induced unconsciousness of the patient with subsequent extinction of reflexes and pain perceptions), regional anaesthesia, and local infiltration anaesthesia [9]. Regional anaesthesia can be divided into spinal anaesthesia, which involves anesthetizing segments of the spinal cord, and peripheral regional anaesthesia (pRA), also known as nerve blocks (PNB). Local anaesthesia is the simplest form of anaesthesia. It is defined as the infiltration of a local anaesthetic, usually lidocaine, directly into the tissue, numbing the skin and surrounding tissue for the procedure [9]. In contrast to local infiltration anaesthesia, pRAs are procedures in which local anaesthetics are injected next to one or more specific nerves, resulting in a temporary interruption of signals transmission, allowing painless surgical procedures in that particular part of the body [9]. Local anaesthesia, on the other hand, targets diffuse nerve branches in the immediate vicinity of the surgical site. It is less effective and of limited use in cases of inflammation in the surgical area [10].

In high-income countries (HIC) with a high density of anaesthesiologists, the choice of anaesthesia depends primarily on the type of surgical procedure and the patient's constitutional situation. In LICs, regional anaesthesia should be preferred over general anaesthesia whenever possible due to a massive shortage of trained anaesthesiologists and physician anaesthetists, inadequate or dysfunctional equipment, and limited availability of drugs needed for general anaesthesia, such as opioids [11, 12]. It is also less likely to have harmful side effects, while the patients maintain spontaneous breathing [13–15]. Bearing in mind that access to appropriate monitoring in case of complications must still be given throughout the procedure.

Overall, regional anaesthesia can improve treatment outcomes, patient safety, and comfort, not only for advanced surgical procedures but also for minor procedures, such as the removal of an infected toenail, a foreign body, or a suture. These procedures are often performed without any form of anaesthesia in LICs [16]. In addition, when operating on an infected or inflamed area, such as an abscess or infected toenail, where local infiltration anaesthesia is not effective due to the acidic milieu, a nerve block can be performed outside the infected area by targeting the supplying nerve and anywhere along its topographical landmarks [9]. Finally, pRAs often provide patients with better intraoperative and postoperative pain management compared to local anaesthesia due to their longer-lasting and more effective pain relief [17].

However, the implementation of pRA techniques in a clinical setting requires appropriate training of medical staff by specialists, which in turn are hardly available in the LICs [18]. In addition, the inadequate supply of certain anaesthetic drugs, such as bupivacaine and lidocaine, may be another barrier to the safe implementation of peripheral regional anaesthesia [19].

The study aims to evaluate anaesthetic practices for surgical interventions at Gulu Regional Referral Hospital in Northern Uganda, focusing on the use of pRA.

The primary objective is to ascertain the circumstances under which pRA procedures are employed in surgical interventions at Gulu Regional Referral Hospital and to identify the factors that influence the utilisation of pRA.

The specific objectives include an analysis of staffing levels and their adequacy in supporting pRA for surgical procedures, as well as a quantification of surgical interventions performed with inadequate anaesthesia, where pRA could have been beneficial. In addition, the study will identify a possible target group for pRA training and develop training content based on the identified needs. In addition, we assessed the number of interventions that were performed with inadequate anaesthesia, where pRA could have been utilised. Our aim was to identify a target group for pRA training and provide information on useful training content.

## Methods

### Study area and population

The study was conducted at the Surgical Department of Gulu Regional Referral Hospital in northern Uganda. The referral hospital serves a population of 374,700 people and is one of the oldest hospitals in the region. It has a capacity of 359 beds [20] and two operating rooms. Patients are entitled to free treatment at a referral hospital. To date, no physician anaesthetist has been employed in the hospital according to available records [21]. Spinal anaesthesia is commonly administered by anaesthetic officers for lower extremity procedures. In 2013, anaesthetic officers accounted for 1.1% (4/352) of the hospital staff [21].

### Study design and data collection

A retrospective data analysis of routine clinical records of the hospital was conducted in February 2020. To assess the potential of pRA implementation, medical record books were screened, and all surgical interventions performed in 2019 in the surgical department were documented: accidents and emergency (A&E), surgical ward, general operation theatre, and orthopaedic surgery theatre. Due to missing data, only the following time periods could be documented for 2019: surgical subunit of A&E from 22/08/2019 to 31/12/2019; surgical ward from 01/01/2019 to 31/12/2019; general operation theatre from 27/05/2019 to 31/12/2019 and in the orthopaedic surgery theatre from 22/08/2019 to 31/12/2019.

Medical record books were handwritten, and data was only included if the documentation was legible. A total of 804 recorded procedures were included in the study, here double entries were excluded in our final data.

For data management we used Memento (Android Client for Memento Database, version 4.7.6), a smartphone-based system with an additional desktop version.

The following data was identified from medical record books: the location of the procedure, the patient's sex and age, the diagnosis, the surgical procedure, the location of the intervention, whether an AO was present (yes or no), the anaesthetic drugs used (if any), the type of anaesthesia (Local Anaesthesia (LA), peripheral Regional Anaesthesia (pRA/PNB), Spinal, and General or Sedation Anaesthesia (GA)), and the provider of surgical care and their qualification (surgeon, medical officer, intern doctor, medical student, or nurse). The latter has been categorised into three different training levels, as shown in Table 2 in the appendix.

1. High level of training = Health worker (HW) with a medical degree and advanced training of more than 2 years (surgeons and medical officers)
2. Intermediate lever of training = HW with a medical degree in their first year of training (intern doctors)
3. Basic level of training = HW without a medical degree (medical students and registered nurses working in A&E, the surgical ward or in OR)

To answer the study question, we retrospectively determined the localization of the intervention (finger, hand, forearm, groin, anus, patella, foot, and toe), as well as the type of intervention (fracture, nail removal, suture, incision or drainage or circumcision), and the absence of infection. We considered that the procedure could have been performed using pRA when all of these conditions were met.

**Fig. 1:**
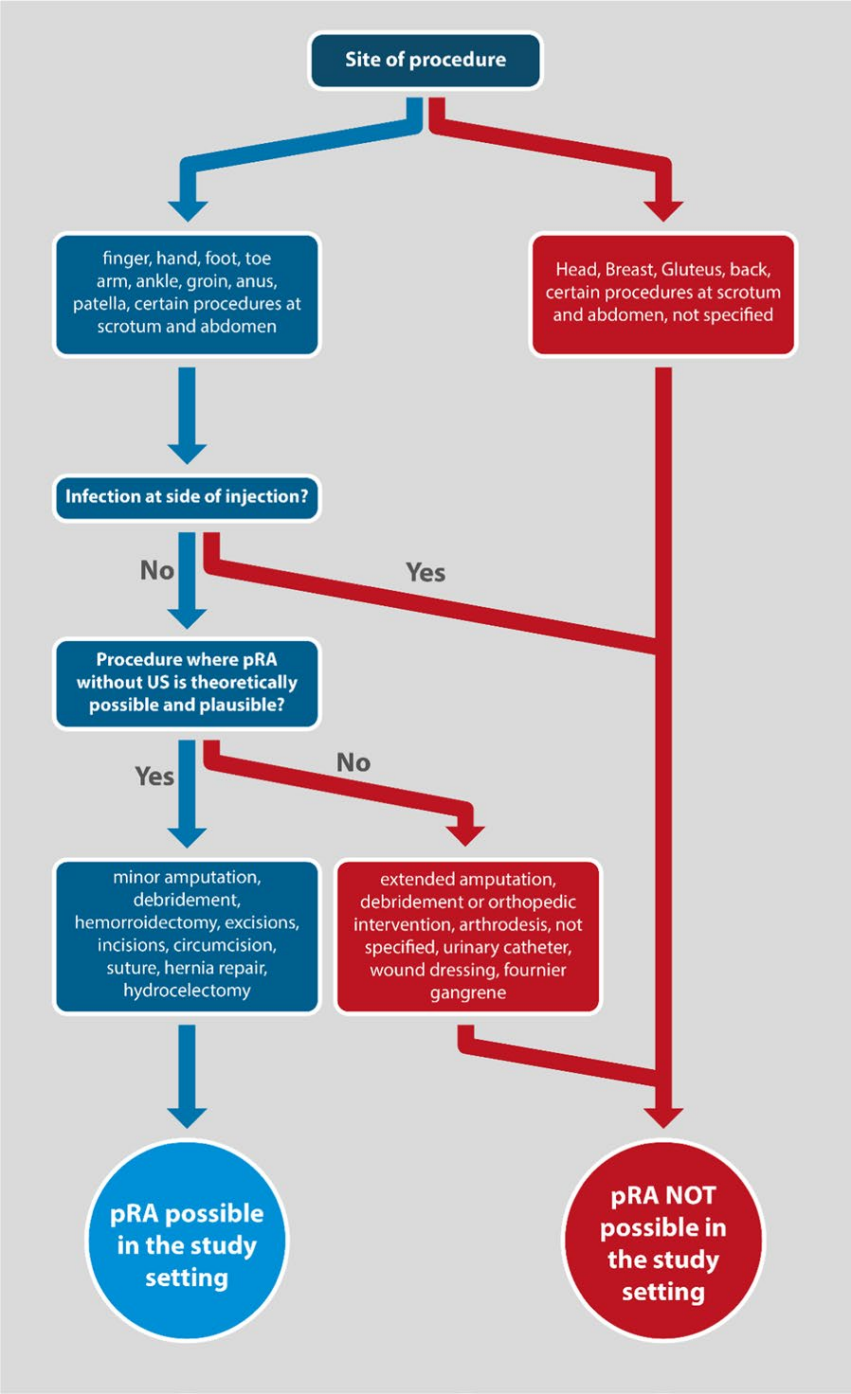
Methodology used to identify surgical procedures suitable for peripheral regional anaesthesia (pRA)

Figure 1 illustrates the methodology used to identify surgical procedures, which would allow pRA.

We checked if pRA was performed for the applicable procedures. If pRA was performed, we considered the anaesthesia adequate. If pRA was not performed or was not performed exclusively, we considered the anaesthesia to be inadequate. Inadequate anaesthesia included the following, as documented in the medical record books: no anaesthesia, general anaesthesia with inadequate pRA, sedation with inadequate pRA, general anaesthesia (where pRA would have been a safer option), and local anaesthesia alone.

### Data safety and ethical consideration

Synchronization and file backup with the computer were done using SyncTrazer and SyncThing clients on the computer and mobile device to guarantee data safety.

In Uganda data was stored on a password secured external hard drive and then transferred to a password-protected server of Charité-Universitätsmedizin Berlin. Data were stored according to data safety regulations of Charité-Universitätsmedizin Berlin and the data safety manual of Institute for General Practice. The study was carried out in line with ICH GCP regulations—Good Scientific Practice of Charité Universitätsmedizin and Gulu Regional Referral Hospital. Data were deidentified and analysed retrospectively. They were retrieved from routine clinical documentation; thus, ethical clearance was not necessary.

### Data cleaning and statistical analysis

Statistical analysis of the questionnaires was performed using SPSS (IBM, version 24). Data were cleaned for duplicates, implausible or missing values, and formatting errors. We collaborated with staff in the respective surgical units to conduct a data review and confirm the accuracy of the information we obtained.

When necessary, only complete data were used for subsequent analysis. All clinical and demographic parameters were summarized in descriptive statistics. Frequency and percentage were used for categorical data and median and IQR for metric data.

For differences in categorical data Chi-square test (*χ*^2^-test) was used. The effect size was estimated with Phi (*φ*) and the threshold for significance was set to *p* < 0.05. Bivariable logistic regression was used to investigate the association between the delivery of peripheral regional anaesthesia, the qualification of medical staff, and the unit within the hospital. Qualification of medical staff and unit were set as the independent variable (Ref = HW with high level of training or A&E). The dependent variable was the use of pRA when it was deemed appropriate for the surgical intervention. A multivariable logistic regression adjusting for covariates was not performed, since we could not identify meaningful further information from record books.

## Results

A total of 813 data entries were recorded. Nine entries were not considered for analysis due to missing data. The remaining 804 entries were used for further statistical analysis. The relative frequency of recorded procedures between the departments are shown in Table 1.

**Table 1:** Procedure distribution

Record book	Recorded procedures *n* (%)
Operating theatre	252 (31.3%)
Accident and emergency department	534 (66.4%)
Ward	18 (2.3%)
Total	804 (100%)

The mean age of all recorded patients was 24 years (SD ± 17), with a minimum of 1 month and a maximum of 89 years. Males accounted for 63.9% of all cases (*n* = 514), 32.6% were female (*n* = 262), and sex was not recorded in 28 patients (3.5%). Anaesthesia was documented in 82% (*n* = 660) of all procedures with two not specifying which type of anaesthesia was used. Of those specifying the type of anaesthesia applied, 86.0% (*n* = 568) had a form of regional (pRA or Spinal) or local anaesthesia documented, with 67.0% being local anaesthesia (*n* = 378). Twenty procedures (2.5%) were conducted with regional or local and general anaesthesia or sedation. The level of sedation was not documented. Eighteen percent (*n* = 146) were done without anaesthesia.

An anaesthetic officer (AO) was responsible for anaesthesia in 24.0% of all cases (*n* = 193). AOs were more frequently present during an operation when HWs with a high level of training operated (OR = 16.39; 95% CI (10.9–25); *p* < 0.001) and when regional or local anaesthesia was applied (OR = 14.26; 95% CI = 8.31–24.46, *p* < 0.001), as shown in Table 2. Spinal anaesthesia was only applied when an AO was present and only in the OR.

**Table 2:** Factors influencing the presence of an AO responsible for anaesthesia (*n* = 773/658/ 568)

	AO responsible *n* (%)	AO not responsible *n* (%)
Level of training of operating HWs (*n* = 773; missings = 31)		
Intermediate level of training	67 (37.2%)	510 (86.0%)
High level of training	112 (62.2%) *	52 (8.8%)
Basic level of training	1 (0.6%)	31 (5.2%)
Total *n* (%)	180 (100%)	593 (100%)
Type of anaesthesia (if anaesthesia was applied) (*n* = 658, missings = 2)		
General or sedation	70 (36.3%)	20 (4.3%)
Regional or local	108 (56.0%)*	440 (94.6%)
General/sedation and pRA or local	15 (7.8%)	5 (1.1%)
Total *n* (%)	193 (100%)	465 (100%)
Type of regional anaesthesia (if regional anaesthesia was applied) (*n* = 568)		
pRA or local	62 (50.4%)	67 (15.1%)
Spinal	61 (49.6%)	0
Local only	0	378 (84.9%)
Total *n* (%)	123 (100%)	445 (100%)

*p < 0.001

**Fig. 2:**
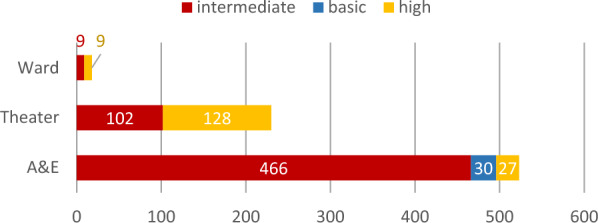
Distribution of procedures according to training level and location within the surgical department (*n* = 773 procedures)

Figure 2 shows the distribution of operating HWs according to their level of training. Within different departments, significantly more interns operated in A&E than in other sites of the surgical department (*p* < 0.001).

**Fig. 3:**
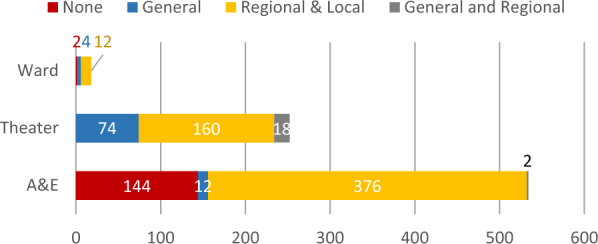
Distribution of procedures according to location within the surgical department and form of applied anaesthesia (*n* = 804 procedures)

For most surgical procedures some form of regional or local anaesthesia (*n* = 548; 68.2%) was used. Anaesthesia was always used during procedures in the OR. Out of the 146 procedures conducted without anaesthesia, 144 (98.6%) were carried out in A&E. Figure 3 displays the distribution of anaesthesia procedures according to the location within the hospital, where the procedure was performed.

Out of 804 recorded procedures, a retrospective classification regarding a theoretically possible peripheral regional anaesthesia (pRA) could be made for 447 entries. Of all registered procedures that underwent the full classification algorithm, 25.9% (209 procedures) would have been possible with pRA. (Fig. 2 in appendix). More than half of those procedures (124; 59.3%) also received a form of pRA. In this subgroup, HWs with a high level of training operated under adequate anaesthesia significantly more often than those with intermediate or basic levels of training. (OR = 2.586; 95% CI = 1.336–5.005; *p* = 0.005).

Of those procedures performed with inadequate anaesthesia, almost half (38, 44.7%) received local anaesthesia alone. Presumably unnecessary general anaesthesia or sedation was used in 20.0% [17]. However, the extent of sedation was not documented. In 17.6% of cases, pRA was inadequate and required additional general anaesthesia or sedation via the administration of ketamine and/or midazolam, and a further 17.6% received no anaesthesia.

In cases where inadequate anaesthesia was administered, the procedure was performed by HWs with an intermediate level of training in 70.7% of cases (*n* = 58). Out of all cases with inadequate anaesthesia, 78.8% (*n* = 67) were performed in A&E.

**Fig. 4:**
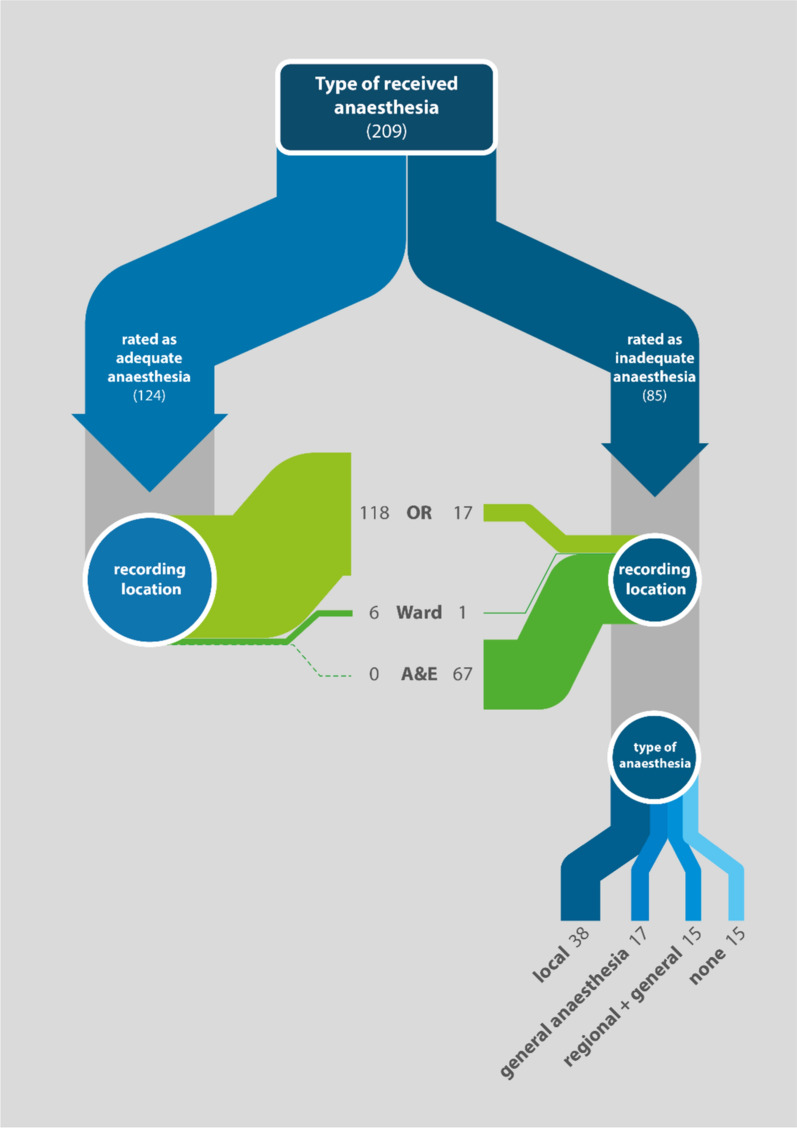
Rated as adequate/inadequate for pRA in relation to setting and type of anaesthesia (209)

HWs with an intermediate level of training never recorded adequate pRA during a procedure in A&E (*p* < 0.001) (Fig. 4). Procedures that would have benefitted from pRA involved minor operations on the hand, foot, and groin.

## Discussion

### Main findings

This study demonstrates that regional anaesthesia procedures are already widely used and accepted practice at the Gulu Regional Referral Hospital in Northern Uganda. They accounted for 86.0% of all forms of anaesthesia administered to surgical patients. However, only one-third of the procedures involved spinal or peripheral nerve blocks (pRA/PNB). The majority were documented as local anaesthesia alone. These are often less effective than nerve blocks due to their smaller volumes and reduced pharmacological effects in inflamed areas, resulting in less effective and shorter-lasting pain relief. Yet, the pharmaceuticals and materials used for local anaesthesia could also be readily deployed in the administration of more efficacious and long-lasting nerve blocks. Consequently, our analysis has demonstrated that the material is available in sufficient quantities. Furthermore, we were able to rule out other possible factors influencing the low level of use, such as costs and a lack of infrastructure or electricity, since the application of the necessary nerve blocks does not require any electricity-dependent equipment.

### Possible reasons for low use of pRA procedures

Interestingly, spinal and nerve blocks were administered significantly more often when HWs with a high level of training were operating and mostly in the main operating theatre. In contrast, the medical records of the A&E department did not identify a single adequate pRA. Although especially simple nerve blocks to the distal extremities would often have been beneficial. Instead, local anaesthesia was more commonly used in this setting. A likely reason for the low implementation of nerve blocks is the severe shortage of anaesthesiologists and non-physician anaesthesia providers in Northern Uganda and Uganda in general. In 2017, Uganda had approximately 400 non-physician anaesthesia providers, while the number of anaesthesiologists was only 60 [5]. Gulu Regional Referral Hospital was no exception in this regard and at the time did not have any trained anaesthesiologist. Instead, they employed four anaesthetic officers (AO) as non-physician anaesthesia providers. However, given the documented number of over 800 procedures in 2019, four AOs are still considered insufficient for the number of surgical procedures conducted at GRRH. As a result, an AO cannot be present for all procedures requiring anaesthesia, and some HWs are often left alone during surgical procedures. Furthermore, it is important to note that the training of an AO is limited and does not encompass all aspects of anaesthesia care, including the administration of nerve blocks or more advanced forms of general anaesthesia. This is due to the duration of the Higher Diploma in Anaesthesia, which is 2 years, compared to a 5-year medical degree plus additional years in an anaesthesia fellowship program [22].

According to our data, spinal anaesthesia was exclusively performed by AOs, while pRA procedures were performed by HWs without an AO in three quarters of cases. The division of labour exists, because pRA is not currently included in the 2-year training program, which only covers spinal anaesthesia as a form of regional anaesthesia [22]. However, certain simple forms of peripheral nerve blocks, such as digital, toe, wrist, and ankle blocks, are easy to learn and do not necessarily require an ultrasound machine if the practitioner has advanced anatomical knowledge. In addition, they allow for surgery over a wider anatomical area than local anaesthesia alone, i.e., interventions below the knee and wrist [23].

One possible explanation for the higher frequency of pRA procedures performed by highly trained HWs, compared to HWs with only 1 year of experience or less, may be the need for sufficient clinical experience. However, it is worth noting that the majority of A&E staff are interns, who have only 1 year of training. A&E serves as the first medical response to accidents, and many minor procedures are performed. In this context, patients with minor wounds, especially if located at the distal extremities, could benefit from pRA as an adequate, safe, cost-effective, and time-efficient pain management tool [23]. Interns in turn would benefit from training in these techniques.

### Comparison with other studies on pain management in LIC

Trauma has been described by the WHO as a hidden epidemic. Road traffic accidents and violence are among the top 10 causes of death and persistent morbidity in low-to-middle-income countries [24]. A study conducted in Ibadan, Nigeria, showed that patients who have experienced traumatic injuries often receive no or inadequate analgesia. In one university hospital, half of the patients with pain in the emergency department did not receive any analgesia at all, and of those who did receive an oral or intravenous analgesic, 80% were left with moderate to severe residual pain [25].

### Implications for policy and training

This and our findings show that we need to look outside the specialised anaesthesia task force to facilitate and spread the division of labour.

Task sharing is already common practice in many emergency departments in high-income countries (HIC), where emergency physicians and anaesthesiologists work jointly to ensure pain-free treatment of their patients [26]. In this specific and similar settings, pRA may be a good alternative to otherwise opiate-based analgesia in the emergency department [27, 28]. It may also reduce the risk of opiate-related complications and adverse effects (including respiratory depression, abuse, nausea and confusion) [27]. Furthermore, as the availability of opioids is still very limited in sub-Saharan Africa, particularly in rural areas, pRAs can play an important role in providing pain relief in LMICs [12].

As other previous studies have shown, task sharing as well as task shifting is already widespread in LMICs and works well across different disciplines but needs to be further strengthened in the field of surgery and anaesthesia [5, 29–31]. However, to adequately address the need and improve patient safety and user satisfaction, and thus better outcomes, a needs-based training programme in pRA procedures adapted to the current local situation and equipment is crucial [32–34]. Our data indicates that interns could benefit most from such a training programme, particularly for simpler nerve blocks. This could improve patient-centred care, especially in A&E. However, the programme should cover all aspects of these nerve blocks, including their typical and atypical anatomical course, indications and contraindications, and possible complications and their management [35].

In a setting such as Gulu Regional Referral Hospital, it is necessary to adopt a low-key approach with minimal technical equipment and requirements for the successful and sustainable delivery of training [36]. Therefore, as well as identifying the target audience, we carefully selected the necessary pRA procedures as training content. Our results showed that blocks for distal parts of the extremities, such as the digital, toe, ankle, and wrist block, as well as the penis block, would be useful in the mentioned setting. The research findings were later integrated into a training application named ‘NoPain UG’, which serves as supplementary educational material [37].

Based on our findings, a training program was devised and subsequently implemented at Gulu Regional Referral Hospital, with the objective of meeting the specific training needs identified. The program was designed to educate a diverse cohort of medical and non-medical personnel on the effective performance and assistance of the aforementioned nerve blocks. This initiative will be accompanied by a research project focusing on the effects of the training measure on patient safety, patient outcomes, and access to anaesthesia and quality of care.

We hope that this approach will support the state strategy short-term by addressing the shortage of anaesthesia providers now. In 2017, a Bachelor of Science in Anaesthesia (BScA) programme was introduced alongside the Diploma in Anaesthesia. The BScA programme takes 3–4 years for non-physicians to complete. We encourage other healthcare workers to enrol in the programme, which takes a long-term specialised approach [38].

### Strengths of the study

A strength of this study is the large amount of data collected for many patients and procedures. We were able to analyse data from 804 procedures over 1 year. Another strength of this study is the comprehensiveness of the patient data used. The procedures selected for analysis were characterised by consistent and legible data, which significantly minimised the potential influence of missing data.

### Limitations of the study

First, a retrospective study can be a source of bias. It is not possible to fully ensure correct categorization of data retrospectively, as it depends on proper and thorough documentation of medical records. The documentation may have been influenced by the requirement to record certain procedural details, resulting in either manipulated, inaccurate or incomplete data, or no documentation at all. Furthermore, the classification of regional anaesthesia procedures might not have been clear to all medical providers, which could have led to over- or under-reporting of certain anaesthesia forms. The distinction between general anaesthesia and sedation was not always clear in the record books, and as a result, they were combined in this manuscript. This may have led to over- or under-reporting of one or the other. However, as the focus of this study is on pRAs, this imprecision has been accepted.

Furthermore, it was not possible to distinguish between the various specialties AOs are required to hold prior to obtaining their diploma, as this information was not documented. However, as this was not the primary objective of this research project, we accepted this lack of clarity and simply included AO as a category. Retrospective subgrouping was based on a combination of information about the location and type of procedures and conditions treated. Therefore, incorrect, or inadequate documentation of this information may have led to inaccurate grouping. However, we collaborated with staff in the respective surgical units to conduct a data review and confirm the accuracy of the information we obtained.

To improve the above limitations in our current study, we have initiated a prospective study on specific nerve blocks while conducting planned trainings at Gulu Regional Referral Hospital.

### Generalizability

The study population is representative of the general patient population in a referral hospital in Northern Uganda, and potentially in other LICs. Our results and methods demonstrate potential for generalisability. The study design could be extended to other hospitals, particularly those lacking evidence on the use of pRA in referral hospitals in LICs.

## Conclusion

Anaesthesia is still a neglected area in the field of medicine in low- and middle-income countries [14]. This study provides an initial overview of the status of regional anaesthesia in a regional referral hospital in Northern Uganda. In this, the use of nerve blocks has many advantages as they can be performed independently by HWs, thus addressing the shortage of qualified physician and non-physician anaesthesia providers. Although nerve blocks are more technically demanding than local anaesthesia and, therefore, require better training to perform correctly, the same equipment is used for both procedures.

The data show that pRA procedures are already being used at the GRRH, especially by HWs with a high level of training and in the operating theatre. It allows us to identify HWs working in the accident and emergency department as the most promising target audience who would benefit from training in this area.

In the A&E department, which is primarily staffed by doctors with less than 1 year of training (mostly intern doctors), there is potential to increase the administration of adequate pRA by introducing simple forms of nerve blocks into routine clinical practice. These blocks do not require ultrasound or continuous monitoring. Therefore, intern doctors, other A&E staff, and ultimately their patients would benefit from needs-based training in nerve blocks, such as digital, toe, ankle, and wrist blocks. However, staff who receive this training should already have basic knowledge of physiology, pharmacology, and anatomy to prevent complications and morbidity. Therefore, interns are suitable candidates for specialized training programmes as they already possess the necessary educational background. However, it is important to include other HWs in A&E to a certain extent. They also require training in preparing for and assisting doctors during procedures, as well as monitoring patients afterward.

By providing physicians in low-resource settings with a specially designed training programme in peripheral regional anaesthesia, our goal is to assist clinics and governments in their task-sharing strategy and ultimately reduce the burden of inadequate anaesthesia on patients.

## Supplementary Information

Additional file 1.

Additional file 2.

Additional file 3.

## Data Availability

The data sets used and/or analysed during the current study are available from the corresponding author on reasonable request.

## Abbreviations

GRRH: Gulu Regional Referral Hospital
pRA: Peripheral regional anaesthesia
AO: Anaesthetic officers
HW: Health worker
A&E: Accidents and emergencies department
OR: Operating room
LICs: Low-income countries
HICs: High-income countries
