# Supplementary figures and images for: Anaesthetic practices at Gulu Regional Referral Hospital in Northern Uganda, who does what and where? A retrospective study
Source: Hum Resour Health. 2025 Apr 14;23:19. doi: 10.1186/s12960-025-00987-4 (PMC11995501; doi:10.1186/s12960-025-00987-4)

Supplementary material: Figure 1

| 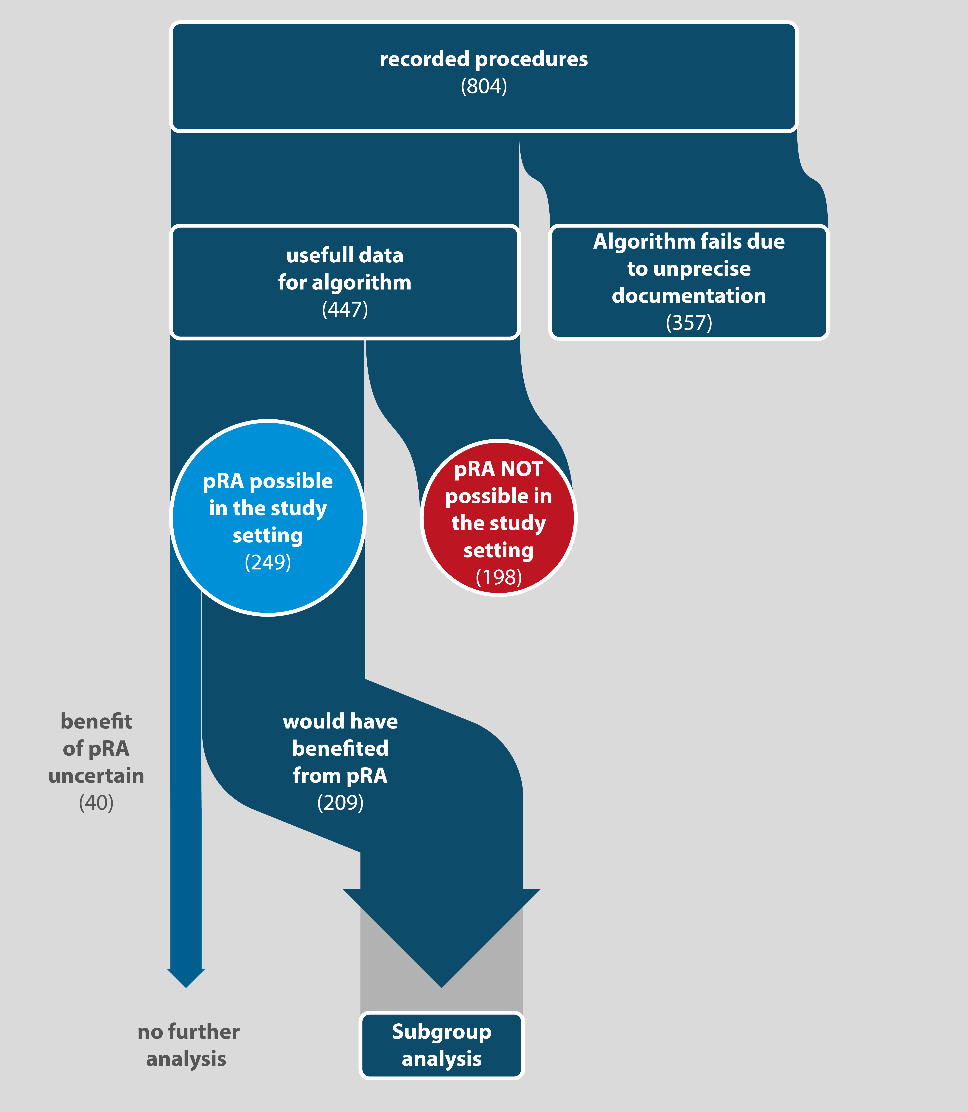 |
| --- |
|  |

Supplement: Supplementary file 1 — Additional file 1. [file 12960_2025_987_MOESM1_ESM.docx]
