# Supplementary material for: Anaesthetic practices at Gulu Regional Referral Hospital in Northern Uganda, who does what and where? A retrospective study
Source: Hum Resour Health. 2025 Apr 14;23:19. doi: 10.1186/s12960-025-00987-4 (PMC11995501; doi:10.1186/s12960-025-00987-4)
Supplement: Supplementary file 2 — Additional file 2. [file 12960_2025_987_MOESM2_ESM.docx]

|  | | **n**  **(%)** | **Sex of the patient** | | **Setting** | | | **Level of training** | | | **RA possible** | | **Type of anaesthesia** | | | | **Anaesthetic officer present** | |
| --- | --- | --- | --- | --- | --- | --- | --- | --- | --- | --- | --- | --- | --- | --- | --- | --- | --- | --- |
|  | |  | **male** | **female** | **theatre** | **emergency room** | **ward** | **intern** | **advanced training** | **basic training** | **no** | **yes** | **none** | **general** | **regional or local** | **general + regional** | **yes** | **no** |
| **Sex of the patient** | **male** | 514  (66.2%) |  |  | 155  (67.4%) | 346  (65.5%) | 13  (72.2%) | 371  (65.8%) | 103  (68.2%) | 22  (68.8%) | 75  (56.0%) | 145  (72.1%) | 86  (59.7%) | 45  (58.4%) | 366  (68.3%) | 17  (89.5%) | 116  (66.7%) | 398  (66.1%) |
|  | **female** | 262  (33.8%) |  |  | 75  (32.6%) | 182  (34.5%) | 5  (27.8%) | 193  (34.2%) | 48  (31.8%) | 10  (31.2%) | 59  (44.0%) | 56  (27.9%) | 58  (40.3%) | 32  (41.6%) | 170  (31.7%) | 2  (10.5%) | 58  (33.3%) | 204  (33.9%) |
|  | **total entries** | 776  (100%) |  |  | 230  (100%) | 528  (100%) | 18  (100%) | 564  (100%) | 151  (100%) | 32  (100%) | 134  (100%) | 201  (100%) | 144  (100%) | 77  (100%) | 536  (100%) | 19  (100%) | 174  (100%) | 602  (100%) |
| **Setting** | **theatre** | 252  (31.3%) | 155  (30.2% | 75  (28.6%) |  |  |  | 102  (17.7%) | 128  (78.0%) | 2  (6.2%) | 50  (34.5%) | 135  (64.4%) | 0 | 74  (82.2%) | 160  (29.2%) | 18  (90.0%) | 193  (100.0%) | 59  (9.7%) |
|  | **emergency room** | 534  (66.4%) | 346  (67.3%) | 182  (69.5%) |  |  |  | 466  (80.8%) | 27  (16.5%) | 30  (93.8%) | 94  (64.8%) | 67  (32.3%) | 144  (98.6%) | 12  (13.3%) | 376  (68.6%) | 2  (10.0%) | 0 | 534  (87.4%) |
|  | **ward** | 18  (2.3%) | 13  (2.5%) | 5  (1.9%) |  |  |  | 9  (1.5%) | 9  (5.5%) | 0 | 1  (0.7%) | 7  (3.3%) | 2  (1.4%) | 4  (4.5%) | 12  (2.2%) | 0 | 0 | 18  (2.9%) |
|  | **total entries** | 804  (100%) | 514  (100%) | 262  (100%) |  |  |  | 577  (100%) | 164  (100%) | 32  (100%) | 145  (100%) | 209  (100%) | 146  (100%) | 90  (100%) | 548  (100%) | 20  (100%) | 193  (100%) | 611  (100%) |
| **Level of training** | **intern** | 577  (74.6%) | 371  (74.8%) | 193  (76.9%) | 102  (44.0%) | 466  (89.1%) | 9  (50.0%) |  |  |  | 98  (69.5%) | 120  (62.2%) | 124  (86.1%) | 33  (38.8%) | 408  (77.5%) | 12  (66.7%) | 67  (37.2%) | 510  (86.0%) |
|  | **advanced training** | 164  (21.2%) | 103  (20.8%) | 48  (19.1%) | 128  (55.1%) | 27  (5.2%) | 9  (50.0%) |  |  |  | 36  25.5%) | 64  (33.1%) | 2  (1.4%) | 52  (61.2%) | 104  (19.8%) | 6  (33.3%) | 112  (62.2%) | 52  (8.8%) |
|  | **basic training** | 32  (4.2%) | 22  (4.4%) | 10  (4.0%) | 2  (0.9%) | 30  (5.7%) | 0 |  |  |  | 7  (5.0%) | 9  (4.7%) | 18  (12.5%) | 0 | 14  (2.7%) | 0 | 1  (0.6%) | 31  (5.2%) |
|  | **total entries** | 773  (100%) | 496  (100%) | 251  (100%) | 232  (100%) | 523  (100%) | 18  (100%) |  |  |  | 141  (100%) | 193  (100%) | 144  (100%) | 85  (100%) | 526  (100%) | 18  (100%) | 180  (100%) | 593  (100%) |
| **RA possible** | **no** | 145  (41%) | 75  (34.1%) | 59  (51.3%) | 50  (27.0%) | 94  (58.4%) | 1  (12.5%) | 98  (45.0%) | 36  (36.0%) | 7  (43.8%) |  |  | 18  (54.5%) | 34  (66.7%) | 90  (35.7%) | 3  (16.7%) | 46  (34.3%) | 99  (45.0%) |
|  | **yes** | 209  (59%) | 145  (65.9%) | 56  48.7%) | 135  (73.0%) | 67  (41.6%) | 7  (87.5%) | 120  (44.0%) | 64  (64.0%) | 9  (56.2%) |  |  | 15  (45.5%) | 17  (33.3%) | 162  (64.3%) | 15  (83.3%) | 88  (65.7%) | 121  (55.0%) |
|  | **total entries** | 354  (100%) | 220  (100%) | 115  (100%) | 185  (100%) | 161  (100%) | 8  (100%) | 218  (100%) | 100  (100%) | 6  (100%) |  |  | 33  (100%) | 51  (100%) | 252  (100%) | 18  (100%) | 134  (100%) | 220  (100%) |
| **Type of anaesthesia** | **none** | 146  (18.2%) | 86  (16.7%) | 58  (22.1) | 0 | 144  (27.0%) | 2  (11.1%) | 124  (21.5%) | 2  (1.2%) | 18  (56.2%) | 18  (12.4%) | 15  (7.2%) |  |  |  |  | 0 | 146  (23.9%) |
|  | **general** | 90  (11.2%) | 45  (8.8%) | 32  (12.2%) | 74  (29.4%) | 12  (2.2%) | 4  (22.2%) | 33  (5.7%) | 52  (31.7%) | 0 | 34  (23.4%) | 17  (8.1%) |  |  |  |  | 70  (36.2%) | 20  (3.3%) |
|  | **regional or local** | 548  (68.1%) | 366  (71.2%) | 170  64.9%) | 160  (63.5%) | 376  (70.4%) | 12  (66.7%) | 408  (70.7%) | 104  (63.4%) | 14  (43.8%) | 90  (62.1%) | 162  (77.5%) |  |  |  |  | 108  (56.0%) | 440  (72.0%) |
|  | **general + regional** | 20  (2.5%) | 17  (3.3%) | 2  (0.8%) | 18  (7.1%) | 2  (0.4%) | 0 | 12  (2.1%) | 6  3.7%) | 0 | 3  (2.1%) | 15  (7.2%) |  |  |  |  | 15  (7.8%) | 5  (0.8%) |
|  | **total entries** | 804  (100%) | 514  (100%) | 262  (100%) | 252  (100%) | 534  (100%) | 18  (100%) | 577  (100%) | 164  (100%) | 32  (100%) | 145  (100%) | 209  (100%) |  |  |  |  | 193  (100%) | 611  (100%) |
| **Anaesthetic officer present** | **yes** | 193  (24%) | 116  (22.6%) | 58  (22.1%) | 193  (76.6%) | 0 | 0 | 67  (11.6%) | 112  (68.3%) | 1  (3.1%) | 46  (31.7%) | 88  (42.1%) | 0 | 70  (77.8%) | 108  (19.7%) | 15  (75.0%) |  |  |
|  | **no** | 611  (76%) | 398  (77.4%) | 204  (77.9%) | 59  (23.4%) | 534  (100%) | 18  (100%) | 510  (88.4%) | 52  (31.7%) | 31  (96.9%) | 99  (68.3%) | 121  (57.9%) | 146  (100.0%) | 20  (22.2%) | 440  (80.3%) | 5  (25.0%) |  |  |
|  | **total entries** | 804  (100%) | 505  (100%) | 262  (100%) | 252  (100%) | 534  (100%) | 18  (100%) | 577  (100%) | 164  (100%) | 32  (100%) | 145  (100%) | 209  (100%) | 146  (100%) | 90  (100%) | 548  (100%) | 20  (100%) |  |  |
