# Supplementary material for: Anaesthetic practices at Gulu Regional Referral Hospital in Northern Uganda, who does what and where? A retrospective study
Source: Hum Resour Health. 2025 Apr 14;23:19. doi: 10.1186/s12960-025-00987-4 (PMC11995501; doi:10.1186/s12960-025-00987-4)
Supplement: Supplementary file 3 — Additional file 3. [file 12960_2025_987_MOESM3_ESM.docx]

Table 2: Qualification of health care providers (1)

| **Specification based on entries in record books** | **Explanation and level of experience** | **Assigned group in the study** |
| --- | --- | --- |
| Nurses | Healthcare professionals who have completed 2.5 years of nursing training. | Basic level of training |
| Students | Currently enrolled in a five-year university programme to become a physician, students are permitted to perform certain procedures during their training. |  |
| Interns | Recent medical school graduates with a provisional license, working for one year under the supervision of senior consultants in surgery, internal medicine, paediatrics, and obstetrics and gynaecology. | Intermediate level of training |
| Medical Officers | physician who has completed their internship but has not yet been able to complete or enrol in a masters programme | High level of training |
| Surgeons | senior consultant with a 3-year master's degree in surgery |  |
| Anaesthetic Officers | Non-physician anaesthesia provider with two years of specialised anaesthesia training in a government programme | Separate group (not included in statistical analysis), indicated if an anaesthetist was present. |

References:

1. Ogei E, Lewis C. Medical Training in Uganda: A Critical but Neglected Part of the Healthcare System. Cureus [Internet]. 6. Juni 2023 [zitiert 9. Januar 2024]; Verfügbar unter: https://www.cureus.com/articles/161834-medical-training-in-uganda-a-critical-but-neglected-part-of-the-healthcare-system
